# Supplementary material for: Soil Factors Key to 3,4-Dimethylpyrazole Phosphate (DMPP) Efficacy: EC and SOC Dominate over Biotic Influences
Source: Microorganisms. 2024 Aug 29;12(9):1787. doi: 10.3390/microorganisms12091787 (PMC11433728; doi:10.3390/microorganisms12091787)
Supplement: Supplementary file 1 [file microorganisms-12-01787-s001.zip › microorganisms-3155543-supplementary.pdf]

Supplementary Materials

Table S1. Primer sequence information.

Genes	Primers	Primer sequence (5'–3')	Fragment size (bp)
AOA_a*moA*	Arch-*amoA*F	STAATGGTCTGGCTTAGACG	635
	Arch-*amoA*R	GCGGCCATCCATCTGTATGT	
AOB_*amoA*	*amoA*-1F	GGGGTTTCTACTGGTGGT	491
	amoA-2R	CCCCTCKGSAAAGCCTTCTTC	
narG	1960m2f	TA(CT)GT(GC)GGGCAGGA(AG)AAACTG	100
	2050m2r	CGTAGAAGAAGCTGGTGCTGTT	
nirS	cd3AF	GTSAACGTSAAGGARACSGG	425
	R3cd	GASTTCGGRTGSGTCTTGA	
nirK	*nirK*-C2F	TGCACATCGCCAACGGNATGTWYGG	448
	nirK-C2R	GGCGCGGAAGATGSHRTGRTCNAC	
nosZ	nosLb	CCCGCTGCACACCRCCTTCGA	302
	nosRb	CGTCGCCSGAGATGTCGATCA	

Table S2. α-diversity index and gene copy number of background microorganisms in different regions.

	TS	SHZ	HZ	DX	SP	HN
			**AOA_*amoA***			
shannon	1.67±0.09 d [#]	2.36±0.05 c	2.65±0.07 ab	2.47±0.08 bc	2.78±0.09 a	0.80±0.15 e
simpson	0.27±0.01 b	0.14±0.01 c	0.12±0.01 c	0.15±0.01 c	0.09±0.01 c	0.65±0.08 a
ace	44.09±5.39 b	46.57±4.70 ab	56.59±3.09 ab	59.62±5.41 a	58.84±8.57 a	17.67±0.48 c
chao	43.71±7.43 a	49.78±9.72 a	54.32±2.24 a	59.78±6.23 a	59.00±9.79 a	15.94±2.28 b
coverage	1.00±0.00 a	1.00±0.00 a	1.00±0.00 a	1.00±0.00 a	1.00±0.00 a	1.00±0.00 a
gene copy number ($\times10^6$)	59.93±3.06 b	28.47±2.06 c	169.19±5.35 a	75.19±4.42 b	61.90±3.67 b	0.07±0.01 d
			**AOB_*amoA***			
shannon	1.66±0.05 b	2.22±0.07 a	2.17±0.06 a	1.48±0.26 b	2.32±0.15 a	0.61±0.15 c
simpson	0.28±0.01 bc	0.17±0.02 cd	0.23±0.03 bcd	0.33±0.08 b	0.12±0.01 d	0.78±0.06 a
ace	41.60±19.02 a	37.30±4.00 a	48.08±3.13 a	24.95±3.13 a	31.76±4.06 a	28.34±11.38 a
chao	35.23±10.84 ab	35.33±2.08 ab	47.54±3.51 a	24.00±2.00 b	33.67±8.15 ab	23.67±8.40 b
coverage	1.00±0.00 a	1.00±0.00 a	1.00±0.00 a	1.00±0.00 a	1.00±0.00 a	1.00±0.00 a
gene copy number ($\times10^6$)	20.91±0.46 a	9.01±0.16 b	5.34±0.16 c	5.08±0.03 c	1.23±0.00 d	0.10±0.01 e
			narG			
shannon	5.55±0.29 c	6.06±0.10 ab	6.26±0.07 a	5.17±0.04 d	6.05±0.09 ab	5.71±0.04 bc
simpson	0.01±0.00 b	0.01±0.00 c	0.01±0.00 c	0.03±0.00 a	0.01±0.00 bc	0.01±0.00 bc
ace	1687.00±137.10 cd	2066.00±61.02 b	2436.00±119.70 a	1617.00±85.08 d	1883.00±41.21 bc	1599.00±69.84 d
chao	1664.00±149.20 cd	2037.00±72.61 b	2364.00±119.90 a	1593.00±59.40 cd	1832.00±41.10 bc	1554.00±77.42 d
coverage	0.97±0.00 ab	0.97±0.00 c	0.96±0.00 d	0.97±0.00 ab	0.97±0.00 b	0.98±0.00 a
gene copy number ($\times10^6$)	9.85±0.08 c	27.11±0.51 a	22.19±0.84 b	8.19±0.59 c	4.35±0.11 d	3.72±0.41 d
			nirK			
shannon	3.55±0.28 ab	3.57±0.37 ab	3.97±0.26 ab	3.82±0.16 ab	4.07±0.18 a	3.36±0.11 b
simpson	0.08±0.03 a	0.11±0.08 a	0.06±0.02 a	0.04±0.01 a	0.04±0.01 a	0.08±0.00 a

ace	287.80±14.85 b	301.90±5.26 b	361.20±17.56 a	195.60±13.48 c	315.80±20.08 b	150.10±19.65 d
chao	283.90±17.71 b	310.20±15.33 ab	358.50±23.89 a	204.60±22.84 c	320.70±26.76 ab	149.50±19.82 c
coverage	0.99±0.00 b	0.99±0.00 b	0.98±0.00 b	0.99±0.00 a	0.99±0.00 b	1.00±0.00 a
gene copy number (×10^6)	325.46±18.35 a	233.60±9.21 b	99.83±6.01 c	209.45±9.29 b	61.27±1.68 c	57.16±3.72 c
			nirS			
shannon	3.84±0.13 bc	3.45±0.02 c	4.75±0.12 a	3.53±0.12 bc	3.88±0.29 bc	3.90±0.15 b
simpson	0.05±0.01 b	0.07±0.01 ab	0.02±0.00 c	0.09±0.01 a	0.06±0.02 ab	0.04±0.01 bc
ace	300.30±14.60 bc	240.00±11.38 cd	533.60±23.13 a	303.30±0.73 bc	331.80±60.80 b	166.10±14.10 d
chao	299.10±14.19 bc	238.80±17.81 cd	528.70±26.20 a	312.20±3.89 bc	331.20±56.43 b	166.90±13.60 d
coverage	0.99±0.00 b	0.99±0.00 b	0.99±0.00 c	0.99±0.00 b	0.99±0.00 b	1.00±0.00 a
gene copy number (×10^6)	30.95±0.33 b	27.60±2.23 bc	113.34±2.24 a	23.28±1.68 c	3.24±0.06 d	2.85±0.11 d
			nosZ			
shannon	4.81±0.08 b	4.71±0.05 b	5.49±0.09 a	4.64±0.05 bc	4.62±0.17 bc	4.35±0.22 c
simpson	0.02±0.00 ab	0.02±0.00 ab	0.01±0.00 b	0.03±0.00 a	0.03±0.01 a	0.03±0.01 a
ace	659.70±32.49 bc	622.00±21.56 c	1105.00±29.66 a	729.10±58.88 b	653.00±35.99 bc	394.30±40.65 d
chao	653.70±50.94 b	632.10±38.44 b	1097.00±44.07 a	730.20±63.16 b	642.70±28.46 b	394.60±42.59 c
coverage	0.99±0.00 b	0.99±0.00 b	0.98±0.00 c	0.98±0.00 b	0.99±0.00 b	0.99±0.00 a
gene copy number (×10^6)	200.44±5.49 a	206.86±13.70 a	163.39±2.66 b	88.31±4.32 c	85.26±3.23 c	198.16±1.94 a

Note: Results were used as mean ± standard error (Mean ± SE), n=3. # Different letters in the table indicate significant differences (P < 0.05).
